# A Review of Biochar from Biomass and Its Interaction with Microbes: Enhancing Soil Quality and Crop Yield in *Brassica* Cultivation

**DOI:** 10.3390/life15020284

**Published:** 2025-02-12

**Authors:** Kritsana Jatuwong, Worawoot Aiduang, Tanongkiat Kiatsiriroat, Wassana Kamopas, Saisamorn Lumyong

**Affiliations:** 1 Office of Research Administration, Chiang Mai University, Chiang Mai 50200, Thailand; kritsana.ja@cmu.ac.th (K.J.); worawoot.aiduang@cmu.ac.th (W.A.); 2 Department of Biology, Faculty of Science, Chiang Mai University, Chiang Mai 50200, Thailand; 3 Department of Mechanical Engineering, Faculty of Engineering, Chiang Mai University, Chiang Mai 50200, Thailand; tanongkiat_k@yahoo.com; 4 Multidisciplinary Research Institute, Chiang Mai University, Chiang Mai 50200, Thailand; wassana.kamopas@cmu.ac.th; 5 Center of Excellence in Microbial Diversity and Sustainable Utilization, Chiang Mai University, Chiang Mai 50200, Thailand; 6 Academy of Science, The Royal Society of Thailand, Bangkok 10300, Thailand

**Keywords:** biochar, biomass utilization, *Brassica* crops, soil health, environmental benefits, sustainable agriculture, SDGs 15

## Abstract

Biochar, produced from biomass, has become recognized as a sustainable soil amendment that has the potential to improve soil quality and agricultural production. This review focuses on production processes and properties of biochar derived from different types of biomass, including the synergistic interactions between biochar and soil microorganisms, emphasizing their influence on overall soil quality and crop production, particularly in cultivation of *Brassica* crops. It additionally addresses the potential benefits and limitations of biochar and microbial application. Biomass is a renewable and abundant resource and can be converted through pyrolysis into biochar, which has high porosity, abundant surface functionalities, and the capacity to retain nutrients. These characteristics provide optimal conditions for beneficial microbial communities that increase nutrient cycling, reduce pathogens, and improve soil structure. The information indicates that the use of biochar in *Brassica* crops can result in improved plant growth, yield, nutrient uptake, and stress mitigation. This review includes information about biochar properties such as pH, elemental composition, ash content, and yield, which can be affected by the different types of biomass used as well as pyrolysis conditions like temperature. Understanding these variables is essential for optimizing biochar for agricultural use. Moreover, the information on the limitations of biochar and microbes emphasizes the importance of their benefits with potential constraints. Therefore, sustainable agriculture methods can possibly be achieved by integrating biochar with microbial management measurements, resulting in higher productivity and adaptability in *Brassica* or other plant crop cultivation systems. This review aims to provide a comprehensive understanding of biochar’s role in supporting sustainable *Brassica* farming and its potential to address contemporary agricultural challenges.

## 1. Introduction

*Brassica* is a genus that is part of the Brassicaceae family and which includes numerous different crops including such vegetables as broccoli (*Brassica oleracea* var. *italica*), cabbage (*B. oleracea* var. *capitata*), cauliflower (*B. oleracea* var. *botrytis*), kale (*B. oleracea*), and mustard (*B. juncea*), as well as oilseeds like rapeseed (*B. napus*), which is the largest oilseed crop behind soybean (*Glycine max* (L.) Merr.) [1,2,3] and is cultivated across more than 120 countries. These crops are cultivated and consumed all over the world because of their excellent nutritional value and economic potential, as well as their ability to adapt and tolerate a wide range of environmental conditions [4]. *Brassica* species are especially highly valued for their bioactive components, such as glucosinolates, phenolics, vitamins, and minerals, which have been demonstrated to provide significant medicinal properties, including antioxidant, anticancer, antimicrobial, anti-inflammatory, antidiabetic, and neuroprotective activities [4,5]. *Brassica* crops’ impressive nutritional profile and the diversity of their culinary applications have resulted in increased demand from consumers, increasing their worldwide economic significance. Furthermore, oilseeds from some *Brassica* species, such as *B. carinata*, *B. napus*, *B. oleracea*, and *B. rapa*, are important sources of vegetable oil and animal feed, both of which play important roles in the global food supply chain [6,7]. However, intensive cultivation of *Brassica* can result in significant depletion of soil nutrients due to the use of chemical fertilizers, and sustainable management practices are required to maintain crop yields. Therefore, it is important to enhance agricultural techniques that improve soil fertility and reduce reliance on synthetic chemicals. One potential solution is the use of biochar produced from agricultural waste, combined with microbes, which play a critical role in nutrient cycling, disease control, and enhancing plant health [8].

Biochar is a highly carbon-rich material produced by the pyrolysis of lignocellulosic biomass such as wood, manures, plant residues, and other agricultural waste under controlled oxygen-limited conditions [9]. Lignocellulosic biomass primarily consists of three main polymers: cellulose, hemicelluloses, and lignin, along with small quantities of pectin, protein, and extractives. Moreover, the elements in biomass commonly include carbon (C), oxygen (O), hydrogen (H), nitrogen (N), calcium (Ca), potassium (K), silicon (Si), magnesium (Mg), aluminum (Al), sulfur (S), iron (Fe), phosphorus (P), chlorine (Cl), sodium (Na), manganese (Mn), and titanium (Ti), and these chemical components can vary depending on the specific natural biomass sources and origins [10]. Biochar is increasingly being recognized for its potential to advance sustainable agriculture, improve soil properties, and support carbon sequestration [11]. The process of converting agricultural byproducts into biochar provides a comprehensive approach to sustainability. Not only does it effectively decrease agricultural waste and recycle essential nutrients back into the environment, but it also improves soil structure, increases soil fertility, and plays a significant role in climate change mitigation by absorbing and storing atmospheric carbon in a stable form [8,12]. Through these mechanisms, biochar provides a valuable, environmentally friendly tool for building resilience in agricultural systems while addressing major environmental concerns. Presently, biochar is widely recognized for its ability to affect soil efficiency and the physicochemical and biological properties of soils, improving the productivity of crops [13,14]. Regarding the increasing focus on sustainable agricultural methods, biochar derived from agricultural waste has been recognized as an environmentally benign solution to soil quality problems and an effective waste management method [15]. Despite its benefits, application can also lead to unintended negative effects on soil and plant quality, such as having a significantly negative effect on the growth of soil earthworms and fungi, causing a delay in flowering in plants, and resulting in a significant reduction in the root biomass of *Oryza sativa* and *Solanum lycopersicum* in the soil [16]. Several previous studies have demonstrated that different types of biochar generated from biomass waste have a significant synergistic effect, with the particular impact varying based on the type of biomass used, the pyrolysis temperature, and the crop type [9].

Moreover, biochar provides a porous substrate that could support beneficial microbes, increasing microbial diversity and activity in soil. Consequently, microbes such as arbuscular mycorrhizal fungi (AMF), *Bacillus* sp., and *Pseudomonas* sp. increase the availability of essential nutrients, promote plant development, and enhance soil structure [17]. When added to *Brassica* cultivation soils, biochar has the potential to generate a synergistic impact, enriching the population of microbes in the soil and enhancing nutrient availability, resulting in improved plant health and more effective crop yields [18,19,20]. Therefore, we hypothesized that the combined application of different biochar from biomass and microbes should synergistically enhance soil quality and consequently improve the yield and economic potential of plant crops. This comprehensive review focuses on different types of agricultural waste-derived biochar. It delves into the physical and chemical properties of biochar, its interactions with microbial communities, and the processes through which it improves soil fertility and crop yield, especially in *Brassica* cultivation. Furthermore, this review focuses on the potential and limitations of biochar application, which represents an excellent resource for improving agricultural production and sustainability through innovative soil management techniques.

## 2. Production and Properties of Biochar from Biomass

### 2.1. Biochar Production Methods and Parameters

The production of biochar involves pyrolysis, which is caused by heating biomass at high temperatures in the absence of oxygen. Biomass is generated from plants or feedstocks, such as purpose-grown energy crops. These materials consist of three major polymers: carbohydrate polymers, namely cellulose and hemicellulose, and aromatic polymers, namely lignin, which combine to provide plants with mechanical strength and rigidity [9]. Cellulose is an important component of biomass. Essential for maintaining the structural integrity of plant cells, it is a linear homopolysaccharide of cellobiose monomers, consisting of glucose monomers linked by β-1,4-glycosidic bonds [21]. In contrast, hemicelluloses are heteropolysaccharides that consist of multiple branched polysaccharides, comprising glycosyl monomers such as glucose, mannose, xylose, and glucuronic acid. These compounds are formed by monomer chains up to 200 monomers long [22]. Furthermore, the composition of hemicellulose varies significantly depending on plant species. Lignin is a complex three-dimensional amorphous polymer composed of three phenylpropane units, namely, p-hydroxyphenyl (H), guaiacyl (G), and syringyl (S). The three units are formed using different ether (COC) or carbon–carbon (C-C) bonds between monomers [23]. These structural components of the plant cell wall play an important role in determining the optimum pyrolysis temperature for biochar production, because they decompose at different temperatures [24]. Hemicellulose, a polymer with branching and short side chains, decomposes at temperatures ranging from 200–320 °C; cellulose, which is thermally stable due to its semi-crystalline chains, decomposes at temperatures ranging from 314–500 °C; and the phenolic structures of lignin break down over a much wider temperature range, from 160 °C up to 900 °C [25], although studies have suggested that the main reaction proceeds in the broad range of 200–500 °C [26,27].

Biochar production can involve a range of methods to convert organic material, primarily biomass, into a stable carbon-rich substance through thermochemical processes such as pyrolysis, gasification, hydrothermal carbonization (HTC), and torrefaction [14,25,27]. During pyrolysis, three main products are generated: a solid product known as biochar, a liquid part called bio-oil, and non-condensable gases referred to as syngas. The relative production amounts of biochar, bio-oil, and syngas are influenced by the biomass properties, the specific type of pyrolysis, and the conditions during the process [27,28]. Table 1 summarizes the pyrolysis process conditions and the associated product distributions. Pyrolysis is the most widely used process, which typically takes place in low or absent oxygen conditions at temperatures ranging from 300–1300 °C [27,29]. In pyrolysis, the main operating parameters are heating rate, pyrolysis temperature, and residence time [27]. Slow pyrolysis is characterized by a heating rate of <1 °C/s, a temperature range of 300–700 °C, and a residence time ranging from a few minutes to several hours or even days. This process is generally optimized for biochar production, yielding 25–35%. Intermediate pyrolysis is a process that lies between fast and slow pyrolysis. It is characterized by heating rate of 1.0–10 °C/s, pyrolysis temperatures range of 500–650 °C, and a 25% yield of biochar. This process typically maximizes the production of liquid products by using high heating rates and short vapor residence times [14,30]. Fast pyrolysis is characterized by a heating rate of 10–200 °C/s, a temperature range of 300–1250 °C, and a short residence time of 0.5–20 s. This process can produce a high biomass yield of about 75% bio-oil, as well as 13% non-condensable gases and 12% solid biochar. Flash pyrolysis is characterized by a heating rate of >1000 °C/s, a temperature range of 800–1300 °C, and a very short residence time of <0.5 s [27,29]. However, when comparing the classes of slow, intermediate, fast, and flash pyrolysis, slow pyrolysis is a more promising alternative than other pyrolysis processes because it can produce higher biochar yields, while intermediate, fast, and flash pyrolysis can convert a wide range of biomass feedstock for higher bio-oil yields [27,29,31,32].

Moreover, gasification differs from the general pyrolysis process. Gasification is a thermochemical process that converts carbon-rich biomass into syngas, also known as synthetic gas, which is a mixture of carbon monoxide (CO) and hydrogen gas (H_2_). The process is generally performed at high temperatures ranging from 700–1500 °C, with a relatively short residence time of 10 to 20 s in a controlled environment with limited oxygen and/or steam. Gasification mostly produces syngas (85%), along with a small amount of biochar (10%) and bio-oil (5%) [25,33,34]. HTC is another thermochemical process that has been shown to be cost-effective for energy-efficient waste management and value-added product recovery. The process occurs in a reactor at moderate temperatures ranging from 180–300 °C, under autogenous (self-generated) pressure, with biomass residence time ranging from 1 to 16 h [35]. In one report, the HTC process produced approximately 50–80% of biochar, 5–20% of bio-oil, and 2–5% of syngas [14]. Lastly, torrefaction is a type of incomplete pyrolysis during which biomasses are converted into solid components at temperatures between 200 and 300 °C, with a residence time of <30 min, a heating rate of less than <50 °C/s, and the pressure of the atmosphere in the absence of oxygen [36].

**Table 1:** Pyrolysis types and products of pyrolysis processes in a variety of conditions. Modified from Qambrani et al. [14], Visser et al. [25], Pahnila et al. [27], Tomczyk et al. [34], Dang et al. [35], Yadav et al. [36].

Process	Temperature (°C)	Residence Time	Heating Rate (°C/s)	Yields %		
				Biochar	Bio-Oil	Syngas
Slow pyrolysis	300–700	hour–days	<1	35	30	35
Intermediate pyrolysis	500–650	10–20 s	1.0–10	25	50	25
Fast pyrolysis	300–1250	0.5–20 s	10–200	12	75	13
Flash pyrolysis	800–1300	<0.5 s	>1000	20	50	30
Gasification	700–1500	10–20 s	10–300	10	5	85
HTC	180–300	1–16 h	–	50–80	5–20	2–5
Torrefaction	200–300	<30 min	<50	80	0	20

Note: HTC; hydrothermal carbonization.

### 2.2. Types and Sources of Biomass Used for Biochar Production

Biomass consists of three main components: cellulose, hemicellulose, and lignin, all of which are the products of photosynthesis and form the structural components of plant cell walls. It is mostly found as lignocellulosic waste, composed of by-products or residues from agricultural, industrial, and forestry processes [10,37]. The world’s population is expanding on a daily basis and has necessitated increased expansion of both the food and agricultural industries. This has resulted in an excessive amount of these types of materials, which produce an enormous quantity of residue annually. Agricultural residues such as straw, husks, and stalks from crops like rice, wheat, and corn are among the most common sources, due to their large-scale availability and low economic value in many regions [37,38]. These materials are frequently left in the field, and if these residues are released into the environment without proper disposal procedures, for example, by indiscriminate burning or discarding in public places, this may cause pollution of the environment, soil contamination, harmful gas, smoke, and dust, and have harmful effects on human and animal health [39,40]. Forestry residues include materials such as sawdust, bark, tree branches, and leaves, which are all produced through logging, thinning, and other forest management activities [38]. These forest residues are also often either burned or left to decompose on site, emitting carbon dioxide and contributing to climate change [41]. To resolve this problem, numerous studies have investigated the use of biomass as an alternate source in a variety of applications, such as animal production, bioenergy production, composites, composting, and enzyme production, as well as biochar production [37]. Several previous studies have focused considerable attention on these adaptable materials for biochar production, primarily due to their abundance, renewability, and critical role in sustainable waste management [42,43]. Table 2 displays an overview of various types of biomass that have been converted into biochar. These residues include materials from a wide range of crop sources, such as almond, canola, corn, oil palm, peanut, rice, soybean, and wheat [13,14]. Each different type of material has distinct elemental compositions that influence its conversion into biochar, which can be conducted by pyrolysis under a wide range of temperatures. These varying temperatures have considerable influence on the properties of the produced biochar, influencing soil improvement characteristics as well as properties for specific agricultural and environmental remediation applications [9,25].

**Table 2:** Overview of various types of biomass used for biochar production.

Biochar Materials	Pyrolysis Temp (°C)	pH	Elemental Composition (%, Mass Based)								Ash (%)	Yield (%)	References
			C	H	N	O	P	K	Ca	Mg			
Almond (*Prunus dulcis*) shell	300–800	6.9–11.7	24.1–89.4	1.0–6.0	0.5–1.0	11.8–42.0	0.02–0.21	0.16–4.89	0.37–6.01	0.01–0.42	3.4–12.8	25.4–65.1	[44,45,46,47]
Apple tree (*Malus domestica*)	300–800	7.0–10.3	62.2–84.8	0.6–5.7	0.3–3.2	5.8–23.7	0.21–1.49	0.57–1.14	12.90–20.89	3.01–5.64	6.7–10.1	28.5–47.9	[48,49,50]
Bambara groundnut (*Vigna subterranea*) shell	450–750	-	48.4–81.0	2.5–5.1	0.4–2.0	15.4–45.9	5.50	1.43–78.57	0.20–3.05	0.07–1.56	16.1–19.7	45.7	[51,52,53]
Bamboo (*Bambusa*)	250–800	5.2–10.3	38.3–88.4	0.9–4.7	0.2–2.6	5.7–38.5	0.24–2.15	0.30–0.52	0.22–0.34	0.14–0.23	3.0–4.7	17.4–73.2	[20,54,55,56,57]
Beech wood (*Fagus sylvatica*) chips	300–700	5.18–7.8	70.0–88.0	1.5–4.2	1.4	15.0	-	-	-	-	1.2–9.8	22.0–31.7	[58,59]
Brazilian pepperwood (*Schinus terebinthifolia*)	300–600	6.6–9.7	59.3–77.0	2.2–5.2	0.1–34.1	0.3–17.7	0.03–0.09	0.10–0.29	0.73–2.59	0.12–0.29	-	28.9–51.5	[54]
Buckwheat (*Fagopyrum esculentum*) husk	350–650	9.2–10.0	70.1–83.9	1.8–4.4	0.9–1.0	13.3–24.4	-	-	-	-	4.0–33.1	28.5–46.3	[60]
Canola (*Brassica napus*) hull	350–850	-	46.5–55.3	3.8–5.1	0.8–2.6	5.8–18.9	-	-	-	-	16.1–40.7	-	[61]
Canola (*B. napus*) meal	350–850	-	51.1–60.7	3.9–5.2	1.5–3.8	11.0–20.9	-	-	-	-	12.1–31.4	-	[61]
Canola (*B. napus*) stalk	250–650	7.2–11.0	41.7–61.9	1.9–5.0	0.9–1.1	34.2–37.4	1.39–4.25	-	-	-		8.7–70.8	[62]
Canola (*B. napus*) straw	300–700	6.4–10.8	54.9–65.7	3.2–3.5	0.04–1.3	–	0.16–0.48	-	-	-	10.7–28.6	9.2–24.4	[63,64]
Coconut flesh waste	350–600		72.7–83.3	1.5–10.0	2.2–3.0	11.0–13.9					4.6–8.2		[65]
Coffee (*Coffea* sp.) ground	500–550	6.9–10.1	68.0	4.0	3.6–3.7	18.0	0.44	1.79	-	-	6.2	-	[20,66]
Coffee (*Coffea* sp.) husk	350–750	9.7–9.9	60.5–66.0	1.6–3.9	-	9.8–19.5	-	-	-	-	12.9–19.6	43.5	[67]
Conocarpus (*Conocarpus erectus*) tree	200–800	7.4–12.9	64.2–85.0	0.6–4.0	0.7–0.9	4.9–26.6	0.84–1.34	0.38–1.15	43.4–67.5	3.43–7.81	4.5–8.6	-	[13]
Corn (*Zea mays*) cob	250–700	8.6–9.9	43.3–87.2	2.1–5.1	0.4–0.9	9.0–51.3	0.11–0.31	0.78–3.01	0.02–0.11	0.06–0.32	6.1–13.3	18.9–27.1	[20,68,69,70,71,72,73]
Corn (*Z. mays*) stalk	400–600	8.5–10.5	44.7–72.4	1.5–5.8	0.5–2.3	2.2–19.9	0.33	1.52	-	-	11.6–2.3	28.3–38.3	[74,75,76]
Corn (*Z. mays*) stover	300–500	7.3–9.8	45.5–78.1	2.1–5.4	0.3–1.5	6.2–42.0	-	0.01–0.44	0.002–0.33	0.002– 0.23	5.7–32.8	17.0–66.0	[68,77,78]
Corn (*Z. mays*) straw	300–700	7.9–11.4	35.9–85.9	1.5–5.0	0.04–2.4	1.9–31.6	0.25–2.51	0.02–4.57	0.003–0.52	0.003–0.79	3.2–60.2	24.9–43.6	[63,79,80,81,82]
Cotton (*Gossypium* sp.) seed hull	200–800	3.5–10.1	51.9–91.0	0.6–6.0	0.6–1.9	5.9–40.5	-	-	-	-	3.1–9.2	24.2–83.4	[83]
Cotton (*Gossypium* sp.) stalk	550	9.6–10.3	64.3–71.9	3.4–4.8	1.1–1.9	11.0–43.9	0.13	0.7	0.2	0.3	8.8–19.1	-	[75,84]
Cotton (*Gossypium* sp.) straw	300–600	8.4	74.8–84.6	2.3	0.6–1.2	12.7–21.2	0.19	2.56–4.08	0.27–1.17	0.40	2.9	30.5	[80,81]
Date palm (*Phoenix dactylifera*)	300–800	8.3–11.5	58.0–74.6	0.9–4.1	0.3–0.5	2.3–20.8	-	2.18–2.71	4.85–8.08	1.53–2.02	14.4–21.4	-	[85]
Eucalyptus (*Eucalyptus* sp.)	350–750	5.9–10.0	67.4–90.9	1.5–5.4	0.4–0.6	5.6–19.5	-	-	-	-	0.7–1.1	28.2–42.5	[67,86,87]
Hemp (*Cannabis sativa*) stalks	400–1000		73.8–89.1	0.2–3.8	0.6–1.3	0.1–14.9	-	-	-	-	6.5–11.3	-	[88]
Hickory (*Carya* sp.) wood	450–600	8.0–9.4	81.8–84.0	2.2–3.2	0.1–0.7	11.1–14.0	0.02–0.04	0.24–0.34	0.59–0.92	0.13–0.18	-	-	[89]
Larch (*Larix kaempferi*) wood	600	-	91.2	2.0	0.1	2.9	-	0.04	0.05	0.01			[90]
Mulberry (*Morus alba*) wood	350–650	10.2–11.1	67.9–80.1	1.6–4.5	1.6–2.2	16.6–25.2	-	-	-	-	7.5–9.8	22.8–37.5	[60]
Oak (*Quercus* sp.) wood	400–800	6.4–9.7	70.5–89.0	0.7–3.6	0.3–0.7	3.3–21.5	0.10	0.20–0.90	0.31–2.7	0.08–0.20	2.9–13.4	-	[48,90,91]
Oil palm (*Elaeis guineensis*) mesocarp fiber	250–600	-	47.1–67.0	1.9–5.0	1.1–1.4	15.4–42.2	-	-	-	-	14.6	-	[70,92]
Oil palm (*E. guineensis*) empty fruit bunches	250–600	-	47.1–67.3	1.9–6.0	<0.1–1.7	18.1–46.0	-	-	-	-	5.3–13.9	-	[70,92,93]
Oil palm (*E. guineensis*) bark	400	7.1	68.9	5.3	0.9	20.8	-	-	-	-	-	-	[86]
Oil palm (*E. guineensis*) kernel shell	250–600	-	51.9–68.8	2.0–5.7	0.5–0.8	12.9–38.5	-	-	-	-	15.6	-	[70,92]
Oil palm (*E. guineensis*) frond	600	-	69.9	1.7	-	11.0	-	-	-	-	17.6	-	[92]
Oil palm (*E. guineensis*) trunk	600	-	66.3	1.6	-	18.2	-	-	-	-	13.9	-	[92]
Peanut (*Arachis hypogaea*) hull	300–600	6.6–9.1	59.3–77.0	2.2–5.2	0.1–0.3	17.2–34.1	0.03–0.09	0.10–0.25	0.73–1.81	0.12–0.29	-	28.9–51.5	[54]
Peanut (*A. hypogaea*) shell	198–700	7.8–11.1	53.5–84.0	0.9–6.1	0.7–2.7	3.3–33.3	0.19–0.26	0.93–2.21	0.56–2.49	0.23–0.41	1.2–24.4	21.9–45.7	[45,69,79,89,94,95,96]
Peanut (*A. hypogaea*) straw	300–700	6.3–11.2	43.6–53.7	-	1.5–3.5	-	0.46–1.16	-	-	-	16.6–34.9	20.1–38.5	[63]
Pear (*Pyrus communis*) tree waste	300–600	8.9–10.1	66.7–75.1	3.5–5.7	2.9–3.2	18.3–24.6	2.00–1.72	-	-	-	-	-	[49]
Pecan (*Carya illinoinensis*) shell	350–700	4.9–7.2	64.5–91.2	1.5 –5.3	0.3–0.6	1.6–27.6	0.03–0.05	-	-	-	1.8–5.2	-	[94,97]
Persimmon (*Diospyros virginiana*) tree	300–600	8.1–9.9	67.6–76.9	2.9–5.5	2.7–3.1	17.4–24.0	0.77–1.63	-	-	-	-	-	[49]
Pigeon pea (*Cajanus cajan*) stalk	400–600	7.9–10.1	76.17–84.87	2.3–4.5	0.2–0.4	11.7–19.2	-	-	-	-	3.1–4.8	-	[57]
Pine (*Pinus* sp.) bark	350–750	7.8–9.9	67.6–86.3	1.2–3.7	-	19.1–28.7	-	-	-	-	7.9–14.5	38.9–59.6	[67]
Pine (*Pinus* sp.) needle	100–700	-	50.87–93.7	0.6–6.2	0.67 –4.1	2.1–42.3	0.11	0.14	0.68	0.11	0.9–18.7	17.7–30.0	[89,95,98,99]
Pine (*Pinus* sp.) nutshell	600	-	89.0	2.2	0.4	6.7	-	0.30	0.02	0.01	1.8	-	[100]
Pine (*Pinus* sp.) woodchip	150–900	5.8–11.4	49.2–91.5	0.5–6.2	0.1–0.6	5.0–43.6	0.02–0.35	0.17–0.82	0.33–9.23	0.06–0.26	1.0–15.4	14.8–79.8	[44,56,93,99,101,102,103,104]
Pine (*Pinus* sp.) sawdust	600–1600	-	88.9	2.5	0.1	4.1	-	0.16	0.28	0.03	4.5	13.0–17.0	[99,100]
Pistachio (*Pistacia vera*) shell	250–650	4.7–8.8	54.2–89.0	1.3–5.9	0.05–0.7	2.7–37.3	-	-	-	-	1.5–4.8	31.6	[96,97]
*Platanus orientalis*	550	8.0–9.0	67.7–79.7	3.7–5.8	0.6–1.5	9.7 19.3	-	-	-	-	3.6–13.2	-	[75]
Poplar (*Populus* sp.) wood	300–800	8.1–8.2	43.8–81.1	1.5–3.9	0.4–2.8	12.3–51.1	0.06–0.20	0.66–0.92	0.96–1.22	0.13–0.16	32.9	20.0–56.0	[102,105]
Rape (*B. napus*) stalk	550	9.5–9.7	64.1–70.1	4.1–7.0	0.6–2.0	7.9–14.7	-	-	-	-	10.1–19.5	-	[75]
Rapeseed (*B. napus*) straw	300–800	-	55.4–65.0	1.3– 5.6	3.3 –3.9	30.1–36.3	-	-	-	-	27.8–45.1	21.0–38.0	[105]
Rice (*Oryza sativa*) husk	350–800	5.1–9.6	38.8–47.8	0.3–5.1	0.2–0.8	2.7–40.4	-	-	-	-	14.5	65.4–94.4	[48,69]
Rice (*O. sativa*) straw	250–900	6.8–11.1	29.2–88.7	0.3–5.1	0.1–1.9	2.6–26.4	0.01–5.42	1.56–4.80	0.49–13.3	0.81–1.13	10.7–52.0	8.8–50.1	[48,62,63,81,100,101,102,103,104,105,106,107,108,109,110,111]
*Salix babylonica*	550	-	66.0–77.2	2.2–4.9	0.4–1.7	10.7–22.1	-	-	-	-	6.4–10.0	-	[75]
Shea nut (*Vitellaria paradoxa*) shell	600	-	61.3	4.7	0.7	32.3	0.08	0.78	0.23	0.14	-	43.6	[53]
Soybean (*Glycine max*) straw	300–700	7.7–11.1	54.1–62.6	-	0.1–3.6	-	0.27–0.72	-	-	-	-	-	[63]
Soybean (*G. max*) stover	300–700	7.3–11.3	68.8–82.0	1.3–4.3	1.3–1.9	15.5–25.0	-	-	-	-	10.4–17.2	21.6–37.0	[95]
Sugarcane bagasse (*Saccharum officinarum*)	300–750	5.0–9.7	57.0–90.5	1.4–5.2	0.3–1.6	4.3–26.7	0.05–0.42	0.15–1.85	0.10–0.91	0.11–0.21	2.0–23.6	26.9–41.5	[59,67,73,76,80,82,89,112,113,114]
Sunflower (*Helianthus annuus*) husk	550	10.3	78.0	3.4	0.6	12.0	-	-	-	-	5.6	-	[65]
Sweet sorghum (*Sorghum bicolor*) stalk	600	-	74.9	2.9	0.5	21.0	0.60	0.16	0.14	0.35	-	27.1	[53]
Teak (*Tectona grandis*) sawdust	400–700	-	71.7–81.6	2.1–4.0	1.2–1.4	14.9–23.2	-	-	-	-	7.5–12.2	26.5–37.4	[115]
Walnut (*Juglans* sp.) shell	250–800	6.0–9.9	24.1–91.6	1.4–5.7	0.5–0.9	1.7–35.0	0.20	0.16	6.01	0.42	1.1–10.1	22.7–40.5	[45,46,58,96]
Wheat (*Triticum aestivum*) stalk	550	9.3–9.5	53.0–58.8	4.2–5.0	0.9–1.2	7.6–14.2	-	-	-	-	21.5–33.1	-	[75]
Wheat (*T. aestivum*) straw	300–800	7.2–9.4	38.5–78.6	1.2–4.6	0.2–2.3	14.6–34.5	0.10–0.34	0.59–3.60	0.11–2.08	0.21–0.69	8.1–46.0	22.8–33.4	[35,65,79,80,84,93,100,109,116]
Willow (*Salix* sp.) wood chip	450–650	7.3–9.8	78.4–84.8	1.1–2.0	0.8–1.0	-	-	0.57	-	0.23	4.3–5.4	-	[117,118]

### 2.3. Properties of Biochar from Biomass

Regarding biochar production techniques and parameters, several variables have a significant impact on biochar production, particularly the pyrolysis technique and conditions. Numerous parameters such as reactor design and conditions, temperature, residence time, heating rate, and pressure, material composition, particle size, and even pretreatment of the material, all play a significant role in determining the final yield and the chemical and physical properties of the biochar produced [9,25,95,99]. These properties, including surface area, pore structure, functional groups, and elemental composition, are crucial for the biochar’s effectiveness in environmental and agricultural applications [24,107]. Several previous studies have shown that pyrolysis temperature is a particularly important factor, with higher temperatures generally leading to a higher surface area and more pronounced changes in chemical composition. For instance, research by Sun et al. [107] found that increasing temperature releases more volatiles, which alters the biochar’s properties. Other studies, such as those by Ahmad et al. [95] and Zhang et al. [119], observed a direct correlation between higher temperatures and increased surface area. Additionally, higher temperatures tend to increase the pH and C content of biochar, though N levels can decrease, as reported by Hossain et al. [120] and Zhang et al. [119]. Therefore, the selection of a suitable pyrolysis temperature is vital for optimizing both the chemical characteristics and yield of the biochar. Overall, the optimum pyrolysis temperature range for producing biochar is considered to be between 300–800 °C. Table 2 summarizes the impact of pyrolysis temperature on various properties of biochar.

For example, biochar produced from almond shells at temperatures ranging from 300 to 800 °C exhibits a high C content of up to 89.4% [44,45,46,47], while bamboo biochar produced under similar temperature conditions shows a substantial variation in yield (17.4–73.2%) [20,54,55,56,57]. Other materials such as coconut flesh waste and corn cob also produce biochar with varying C content and yields, with coconut biochar reaching 83.3% C but a lower biochar yield (4.6–8.2%) [65], compared with corn cob biochar that had a C content ranging from 43.3–87.2% and a yield between 18.9 and 27.1% [20,68,69,70,71,72,73]. The pH of the produced biochar can also vary widely, ranging from acidic (around 3.5 for cotton seed hull biochar) [83] to strongly alkaline (up to 12.9 for conocarpus tree biochar) [13]. These variations demonstrate the potential for adapting biochar properties to suit specific applications, since its different physical structures affect soil properties such as porosity, water-holding capacity, organic content, bulk density, pH, CEC, and the microbiome. Furthermore, it has considerable environmental benefits with respect to methane emissions, carbon sequestration, nitrous oxide emission, and the sorption of chemicals and heavy metals, according to both the selection of feedstock and the conditions under which it is produced [121]. For example, Jatuwong et al. [20] showed that applying biochar derived from bamboo, corncob, and coffee grounds and produced via a pyrolysis process at 500 °C increased soil pH from 6.31 to slightly alkaline, ranging from 7.44–7.86, during the growth of Chinese kale. This alkaline effect shows the potential of biochar to ameliorate acidic soils and enhance crop growth. Similarly, the simultaneous application of 2% (*w*/*w*) corncob biochar produced at a pyrolysis process of 450 °C increased soil organic matter and available P content by 36.7% and 45.5%, respectively, compared with untreated soil [122], revealing the synergistic effects of biochar on nutrient availability and soil health. Moreover, Devereux et al. [123] observed that adding 5% (*w*/*w*) biochar produced from wood decreased the average pore size in soil from 0.07 mm^2^ to 0.046 mm^2^, which suggested improved soil structure and potential benefits for water retention and root penetration. However, contrasting findings have also been reported. For instance, Basso et al. [124] found no significant change in the CEC of sandy soil after the application of 3% and 6% (*w*/*w*) hardwood-derived fast pyrolysis biochar.

## 3. Biochar and Microbial Interactions for Enhancing *Brassica* Crop Productivity

This summary focuses on the interactions between various biochar materials, microorganisms, and specific *Brassica* crops, highlighting their synergistic effects on plant growth, yield, nutrient uptake, and stress mitigation, as shown in Table 3.

Biochar derived from diverse organic materials such as bamboo, corncob, rice straw, and wood waste has been shown to enhance plant growth and physiological characteristics when combined with specific microorganisms. For instance, bamboo biochar enriched with AMF significantly influenced the growth and chlorophyll content of *B. oleracea* var. *alboglabra*, as reported by Jatuwong et al. [20]. Similarly, the same fungi improved various growth parameters in *B. napus*, including plant height and biomass, and demonstrating a reduction in Cd content and oxidative stress markers, according to Yin et al. [125]. Using corncob biochar in conjunction with bacterial species such as *Ochrobactrum* sp. and *Bacillus mucilaginosus* facilitated increased soil fertility and nutrient content, promoting the growth of *B. rapa* [122]. Furthermore, *Serratia* sp. and *Pseudomonas* sp. bacteria improved drought tolerance and physiological responses in *B. napus* under stress conditions [28]. Rice straw and other agricultural residues, like soybean straw and peanut shells, when combined with bacteria such as *Arthrobacter defluvii* and *Burkholderia cepacia*, have been shown to enhance growth and nutrient uptake in *B. napus* [127]. In particular, sugarcane bagasse biochar led to improved physiological growth parameters for *B. napus*, with increased plant height and yield [76]. Spent mushroom substrate (SMS) has also yielded positive results, with studies showing enhanced growth and biochemical parameters in *B. oleracea* var. *botrytis* through the application of *Bacillus subtilis* and *Pseudomonas fluorescence* [128]. Similarly, maize straw biochar and specific bacteria promoted growth in *B. chinensis*, reducing Cd accumulation due to Cd being evenly distributed and adsorbed on the surface of biochar, thereby reducing its tendency to translocate to plants, while also improving uptake of N and P [18]. Other organic waste materials including paper and pulp waste effectively mitigated cadmium stress in *B. napus* by improving root and shoot growth, photosynthesis rates, and overall plant health [129]. In addition, waste wood from *Morus alba* supported improved water use efficiency and antioxidant enzyme activities in canola plants, enhancing drought tolerance [130].

Moreover, the use of peach residues alongside bacteria like *Pseudomonas putida* significantly improved root and shoot growth in *B. napus* by alleviating oxidative stress and enhancing nutrient uptake, ultimately increasing seed production and oil yield [132]. In *B. chinensis*, the application of *Azospirillum brasilense* led to notably decreased concentrations of heavy metals and improved overall biomass and antioxidant levels in stressed plants [133]. In summary, the application of various biochar materials in conjunction with beneficial microorganisms demonstrates significant potential for enhancing plant growth, nutrient uptake, and resilience to environmental stresses in *Brassica* vegetables. This synergy not only improves agricultural productivity but also contributes to sustainable farming practices by utilizing organic waste materials effectively.

## 4. Environmental and Economic Benefits of Biochar Application

Numerous studies of *Brassica* cultivation systems have demonstrated the significant environmental and economic advantages of applying biochar derived from lignocellulosic materials, such as canola straw, woody waste, and rice husk to crops like cauliflower (*B. oleracea* L.), cabbage (*B. chinensis*), and Chinese cabbage (*B. oleracea* L.) [134,135,136]. Environmentally, biochar enhances soil health by improving water retention, nutrient availability, and microbial biodiversity, while its high carbon stability supports long-term carbon sequestration, reducing greenhouse gas emissions and contributing to climate change mitigation [134,137]. Furthermore, biochar’s ability to immobilize heavy metals and reduce nutrient leaching plays an essential role in reducing soil and water pollution, thereby promoting chemical-free and sustainable agricultural practices [121]. The immobilization of harmful microorganisms and heavy metals via biochar has been recognized as a promising strategy to mitigate soil contamination and enhance agricultural productivity. This capability is attributed to biochar’s high surface area, porous structure, and numerous functional groups, which enhance the adsorption and stability of heavy metals. As a consequence, the bioavailability of these metals in the soil is significantly reduced, decreasing the risk of plant uptake and environmental contamination [95]. Additionally, biochar’s high cation exchange capacity (CEC) and surface area enhance its ability to retain essential nutrients, preventing leaching and improving soil fertility. Biochar’s nutrient-retention properties decrease the risk of nutrient leaching into water bodies, thereby solving problems associated with eutrophication [121,138]. The results of several studies consistently show that biochar application can increase the CEC of soil. Zhang et al. [139] reported that a significant (21%) increase in CEC was seen after the application of rice-husk biochar, in comparison with unamended soil (control). Likewise, El-Naggar et al. [140] found that applying biochar derived from rice straw, silvergrass residue, and umbrella trees increased the CEC of sandy soil by 906%, 180%, and 130%, respectively, compared with unamended soil. Biochar’s high CEC helps it to bind to nutrients and prevents water leaching [121].

Economically, producing biochar from agricultural residues or waste biomass offers a cost-effective solution by reducing dependency on synthetic fertilizers and enhancing input efficiency. This not only decreases production costs but also boosts safe crop productivity and quality, providing economic incentives for farmers [15]. According to a 2023 report, the cost of fertilizers varied significantly, ranging from USD 1153 to USD 1600/ton [141], while the production costs for biochar showed differed significantly, reported at between USD 670 and 17,800/ton, depending on factors such as biomass type, technical processes, and location [142,143]. A global comparison of biochar production costs across continents reveals substantial differences. For instance, in the Americas, biochar production costs can reach as high as USD 17,800/ton. Conversely, in Australia, production costs are reported to be as low as USD 690/ton. In Asia, countries such as China, India, Korea, the Philippines, and Thailand exhibit a broad cost range, from as low as USD 100 to as high as USD 15,400/ton [142,144]. This variation in biochar production costs is mostly due to factors such as biomass type and price, production processes, electricity usage for pretreatment (such as drying and size reduction) and pyrolysis, modifying agents, and biomass storage and transportation costs for both feedstock and biochar, along with remuneration including wages [142,145]. According to Kumari et al. [145], biochar production may be acceptable if revenues derived from those values exceed the economic costs of cultivating, harvesting, shipping, and storing the raw biomass materials, as well as the costs of the pyrolysis, transportation, and use of biochar. Essentially, the overall cost from feedstock to soil application is determined mostly by the costs of the initial biomass resources used and the final production process. Consequentially, utilization of lower-cost biomass and promising processing techniques deserves to be considered, since these might increase the net margin of biochar production [145]. Furthermore, a report found that biochar-based fertilizer is considerably lower in cost than commercial chemical P and K fertilizers. According to Pandian et al. [144], the cost of cotton straw/bentonite biochar-based fertilizer is USD 206/ton, whereas chemical P and K fertilizers cost much more at USD 830/ton. Likewise, the application of biochar has been shown to significantly enhance economic return in cultivation. Recent findings by Zhang et al. [146] revealed that applying biochar at a rate of 3 t ha^−1^ with 70% of the recommended dose of inorganic fertilizer led to a significantly enhanced economic return in pepper cultivation, resulting in USD 9597/hectare. This indicates a significant increase compared with conventional fertilization procedures, which achieved economic returns of USD 6493/hectare. These data highlight the economic feasibility of integrating biochar into long-term *Brassica* cultivation systems. Moreover, its integration into agricultural systems supports the circular economy by converting agricultural waste into valuable products, thereby enhancing economic resilience in farming communities [147]. These combined environmental and economic benefits underscore biochar’s role as a multifunctional, sustainable tool for improving soil quality and crop performance while lowering reliance on chemical treatments in *Brassica* farming systems.

## 5. Potential and Limitations

**Figure 1:**
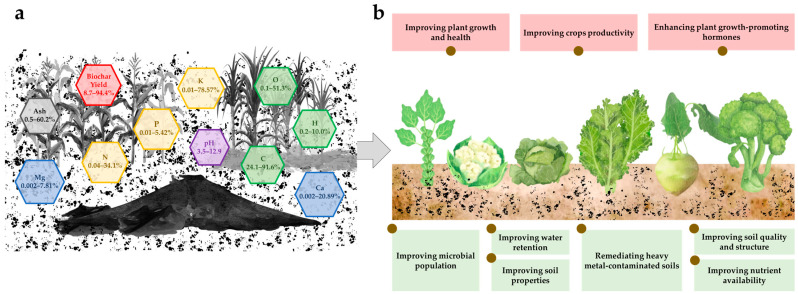
(**a**) Properties and yield of biochar and (**b**) the potential of biochar for environmental remediation and agricultural improvement in *Brassica* crop systems.

The use of biochar and microorganisms presents significant potential for environmental remediation and agricultural improvement (Figure 1 and Table 4). Biochar has been recognized for its ability to remediate heavy metal-contaminated soils by reducing the uptake of harmful chemicals in plants, including heavy metals like Zn, Cd, Pb, and various organic pollutants such as polycyclic aromatic hydrocarbons (PAHs) and brominated flame retardants (PBDEs) [11,42]. This material also enhances soil properties by improving its porosity, reducing density, and promoting better water retention, infiltration, and stability. Such improvements not only combat erosion and mitigate drought but also enhance groundwater quality [148]. Additionally, biochar modifies soil chemical properties, including pH and nutrient availability, while positively influencing biological properties by increasing microbial populations and enzymatic activity [149,150]. Consequently, the application of biochar leads to enhanced crop yields through improved seed germination, root density, and overall growth parameters, ultimately contributing to better agricultural productivity. Furthermore, it aids in carbon sequestration by reducing emissions of greenhouse gases, particularly carbon dioxide and methane.

Microorganisms such as bacteria, fungi, and algae complement biochar’s effects by promoting plant growth, increasing seed germination rates, and improving biomass accumulation. They enhance plants’ tolerance to various stresses, including salinity, drought, and heavy metal exposure, while boosting nutrient uptake [151]. Additionally, some microorganisms protect plants from soil-borne pathogens, with specific strains providing resistance against diseases like *Fusarium* wilt and root-lesion nematodes [152,153]. They also play a crucial role in soil remediation and improvement by producing beneficial compounds such as indole acetic acid (IAA) and siderophores, along with various enzymes that facilitate nutrient availability and microbial community structuring [151].

**Table 4:** Potential and limitations of using biochar and microorganisms.

Impact	Biochar ^a^	Microorganisms ^b^
Potential	- Remediation of heavy metal contaminated soils; Reduced uptake of various chemicals in plants, such as Zn, Cd, fomesafen, PAHs, polybrominated diphenyl ethers (PBDE), arsenic (As), copper (Cu), (chromium) Cr, Ni, Pb, manganese (Mn), methylmercury, sulfamethazine, cesium isotope, carbamazepine, chlorpyrifos, CeO_2_ nanoparticles; - Influence on wettability of soil, water infiltration, water retention aggregation and stability, thereby helping in combating erosion, mitigating drought and nutrient loss, and enhancing groundwater quality; - Influence on soil properties: ○ Improves porosity and thereby reduction in density of soils; ○ Influences wettability of soil, water infiltration, water retention, aggregation, and stability, thereby helping in combating erosion, mitigating drought and nutrient loss, and enhancing groundwater quality; ○ Influences aggregation of soil particles; ○ Improves water quality, soil moisture retention, and its availability to plants; ○ Increases water infiltration, reduces water runoff and thereby erosion of soil particles; ○ Improves soil to granular/crumb structures that are highly suitable for agriculture, with a significant effect on soil texture; ○ Influences soil chemical properties [pH, electrical conductivity, organic carbon, CEC, soil available nutrients (N, P, K, and Al)]; ○ Influences soil biological properties such as microbial population, microbial biomass carbon, soil enzymatic activity; - Benefits to crop yield: Improved seed germination, root density, crop growth, and agricultural yield; - Useful in C utilization as well decreasing gaseous emissions; - Decreased carbon dioxide and methane emissions; Enhanced sequestration of carbon dioxide.	- Promoting plant growth; Enhanced seed germination ratio, seedling length and dry and fresh weight, and plant growth parameters such as number of leaves, fresh and dry weight, plant height, stem diameter, and fruit yield; Plants increased their tolerance to salt stress, drought stress, and heavy metal (Zn, Cd, Cu, Fe, Pb) stress; - Increased Cu concentration in tissues; - Improved protein, phenolics, flavonoids, N, chlorophyll content; - Increased nutrient uptake such as N P, K, Mn, Zn, and Fe; - Protection against soil-borne plant pathogens: *Methylobacterium* sp. 2A protected against *Phytophtora infestans*, *Botrytis cinerea*, and *Fuasrium gramiearum* in *Arabidopsis thaliana*, *Solanum tuberosum*; *Enterobacter cloacae* protected against *Fusarium* wilt (*Fusarium oxysporum*) in *Spinacia oleracea*; *Streptomyces* sp. protected against root-lesion nematode (*Pratylenchus penetrans*) in *Medicago sativa*; *Fusarium equiseti and Glomus mosseae* protected against anthracnose (*Colletotrichum orbiculare*) and damping off (*Rhizoctonia solani*) in *Cucumis sativus*; - Remediation of degraded soils; - Production of IAA; - Production of siderophore - Enzyme activity: 1-aminocyclopropane-1-carboxylic acid (ACC), protease, amylase, pectinase, xylanase, peroxidase/catalase; - N fixation and NaCl, P, Zn solubilization; - Influence on the structure of the microbial community; - Improved soil characteristics and water retention; - Greenhouse gas mitigation
Limitations	- Inhibitory effect on soil aging: negative effects on growth of earthworms and/or fungi; - Aged biochar led to reduction in underground root biomass of *O. sativa* and *S. lycopersicum* in soil; - Using biochar at relatively high rates of 15 t ha^−1^ led to a 200% increase in weed growth during lentil culture; - Technical and practical barriers: Biochar dust, similar to other dusts like coal, woods, foods, plastics, and some metals, can become a combustible hazard; - Combustion risk: Biochar dust can combust spontaneously, posing minimal hazards in handling, transport, and storage in enclosed spaces. This risk needs to be carefully managed to avoid accidents; - Toxic materials: The levels of toxic substances in biochar depend on factors like feedstock type, the production system, and pyrolysis temperature. Many countries regulate exposure to these toxins with a standard “permissible exposure limit”. Thus, there is still no simple and straightforward permissible exposure limit available; - Polycyclic aromatic hydrocarbons (PAHs): During biochar production, especially under pyrolysis conditions, different types of toxic and hazardous PAHs are produced. Biochar application with a high level of PAHs increases the PAH content in plants and soil; - Particle emissions (PM_10_): Biochar-amended soils can generate significant amounts of PM_10_ (particles < 10 μm) and can increase levels of PM_10_ in soil; - PAH composition: The dominant PAHs produced from deciduous/coniferous wood, elephant grass, and wood residue biochar pyrolyzed at high temperatures include phenanthrene and naphthalene; - Cytotoxic effects of fine particles: Fine-particulate biochar poses cytotoxic risks, particularly for human and mouse cells.	- Some IAA-producing bacterial strains can enhance plant root and shoot growth, but have also been shown to inhibit growth at high IAA concentrations; - *Pseudomonas aeruginosa* is an effective strain for degrading hydrocarbon material, but it is also an opportunistic pathogen that causes certain infections like bloodstream, skin, and soft tissue infections, otitis exterma, and pneumonias; - Cyanide-producing bacteria has an inhibitory effect on plant growth, i.e., cyanide production by *Pseudomonas* sp. caused the growth inhibition of lettuce and barnyard grass.

Note: ^a^ All information has been modified from Premalatha et al. [149], Nepal et al. [150], Kavitha et al. [154], Agarwal et al. [155], Das et al. [156], and Bo et al. [157]. ^b^ Microorganisms including bacteria, fungi, and algae; all information has been modified from Antoszewski et al. [151], de Araujo Avila et al. [158], O’Callaghan et al. [159], and Nadeem et al. [160].

Despite the reported benefits, the use of biochar and microorganisms has certain limitations (Table 4). For instance, aged biochar undergoes changes over time due to aging processes, experiencing a reduction in ash content and increased acidity through chemical oxidation, resulting in a lower pH and the release of ash minerals. In contrast, physical aging causes only minor pH changes [161]. However, aged biochar can negatively affect soil organisms including earthworms and beneficial fungi and it may lead to decreased root biomass in some crops such as rice and tomatoes. Biochar is typically produced from agricultural residue, animal waste, or sewage sludge, which can introduce contaminants and contain heavy metals, organic pollutants, toxic chemicals, or hazardous substances into the soil, causing environmental risks or affecting crop health [162,163]. Devi and Sahora [162] and Qin et al. [164] reported that heavy metals such as Pb, Cd, and Zn in biochar can negatively impact microbial growth and reduce biomass while affecting microbial enzyme activity. As a consequence, rigorous characterization of heavy metal concentrations in lignocellulosic waste is essential before its use for biochar production [93]. There are also concerns regarding the presence of toxic materials in biochar, particularly the production of harmful PAHs and volatile organic compounds (VOCs) during its pyrolysis, which can occur as intermediates or by-products from the thermal degradation of lignin, a primary component of lignocellulosic biomass [165]. These compounds can accumulate in soils and plants, presenting potential health risks. PAHs and VOCs have been found to have mutagenic effects on microorganisms, inhibiting microbial activity and altering microbial community structure [165,166]. Additionally, high rates of biochar application can significantly promote weed growth [153]. Practical challenges include the risk of biochar dust combusting spontaneously, posing a hazard during handling and storage. Biochar can contribute to fine particle emissions, which can have cytotoxic effects on living organisms [167]. Therefore, the use of biochar in agriculture raises concerns regarding its limitations, one of which is the difficulty of applying powdered biochar to fields due to its fine, small particles, brittleness, and low density, which cause it to be easily blown away by the wind, resulting in uneven distribution and reduced effectiveness [168]. To reduce the formation of dust and associated loss of biochar, the biochar can be moistened with water or mixed with substances such as manure, sludge, or compost before being used in the field [169]. Furthermore, biochar-induced dust emissions may raise long-term health concerns among users, especially during field application. In order to resolve these concerns, it is critical to explore and develop alternate biochar formulations that facilitate safer, more convenient, and efficient application and lower-risk solutions. For instance, converting biochar into more user-friendly forms such as granules, pellets, other dust-free types, or liquid biochar fertilizers can significantly reduce the risks associated with airborne particles [170]. Microbial strains can also present challenges; while some can enhance plant growth, high concentrations of indole acetic acid may inhibit growth instead. Additionally, certain opportunistic pathogens such as *Pseudomonas aeruginosa* can pose risks to plant and human health [160]. Therefore, careful management and consideration of biochar’s characteristics and microbial interactions are essential to harness its benefits while minimizing potential risks. Overall, while biochar and microorganisms present substantial opportunities for enhancing soil health and agricultural productivity, their application must be approached with caution to mitigate risks associated with their use.

## 6. Future Perspectives and Interesting Driving Issues

The utilization of forestry biomass including leaf litter, branch litter, and various types of plant waste presents a promising pathway for sustainable development, particularly for addressing environmental challenges such as forest fires, biomass burning, and PM2.5 pollution. Converting these types of waste into biochar not only offers a solution to these pressing issues but also creates valuable agricultural materials that can significantly enhance soil health and crop productivity [171]. The development of biochar production from forestry biomass aligns with the principles of the circular economy, turning waste materials into high-value products while reducing greenhouse gas emissions associated with open burning. This approach can also serve as a preventive measure against forest fires by systematically removing excess biomass from forest floors, thus reducing the fuel load and minimizing fire risks [172]. This is particularly crucial during the dry season at the beginning of each year when wildfires frequently occur across tropical regions, including parts of Southeast Asia, Central America, northern South America, and northern Africa [173]. Furthermore, integrating biochar into agricultural systems could lead to enhanced carbon sequestration, contributing to climate change mitigation efforts.

Modifying the properties of biochar depending on the specific requirements of different soil types and crops might help it achieve its full potential in terms of increasing the productivity of economically important crops like rice and maize, as well as cash crops like coffee and rubber. Additionally, exploring the co-benefits of biochar in improving soil microbial activity, reducing soil acidity, and enhancing crop resilience to pests and drought could further solidify its role in sustainable agriculture. On a larger scale, the establishment of biochar production facilities in regions affected by forest fires and biomass waste could stimulate local economies, provide sustainable employment, and support community-based projects. Collaborations between governments, researchers, and the private sector could foster innovation and develop policies that encourage the adoption of biochar. By addressing concerns about environmental pollution and also agricultural productivity, the use of forestry biomass for biochar production has the potential to result in significant advancements in environmental management and sustainable agriculture.

## 7. Conclusions

This review focuses on the important role played by biochar, derived from biomass, in improving soil quality and crop yield, with a focus on *Brassica* cultivation. The characteristics of biochar, such as surface area, pore structure, and nutrient content, are heavily influenced by the production process, which includes varying pyrolysis temperatures. Optimal production conditions, typically between 500 to 800 °C, have been shown to yield biochar that significantly enhances soil health and supports plant growth. The interaction between biochar and beneficial microorganisms has proven essential for boosting the growth and resilience of Brassicaceae crops. Numerous studies have previously indicated that biochar sourced from materials like rice straw, maize stalks, and sugarcane bagasse, when combined with beneficial microbes (e.g., *Pseudomonas* sp., *Bacillus* sp.), enhances nutrient uptake, reduces heavy metal stress, and increases drought tolerance. These synergistic effects contribute to higher crop productivity and promote sustainable farming practices. The environmental and economic advantages of using biochar in agriculture are also notable. It improves soil water retention, nutrient availability, and microbial diversity, contributing to long-term soil health and C storage. Additionally, its ability to immobilize heavy metals and reduce nutrient leaching aligns with eco-friendly farming approaches. Economically, biochar production offers a viable alternative to synthetic fertilizers, with production costs varying from USD 670 to USD 17,800/ton depending on feedstock and methods. This underlines its economic feasibility and supports principles of circular economy that encourage the use of waste materials. While biochar offers numerous benefits, there are challenges to consider. Aged biochar may negatively impact soil organisms, and high biochar concentrations can promote weed growth. Safety concerns, including potential harmful byproducts like PAHs and biochar dust, must also be addressed. Additionally, while some microbes enhance plant growth, excessive concentrations or the presence of harmful pathogens may lead to negative outcomes. To maximize the benefits and mitigate risks, a balanced approach to biochar and microbial management is crucial. Future research should focus on refining production techniques, determining optimal application rates, and identifying the most effective microbial strains for specific soil and crop types. By addressing these factors, the integration of biochar and microorganisms can significantly advance sustainable and productive agricultural practices.

## Figures and Tables

**Table 3:** Summary of the interactions of different biochar materials, microorganisms, and specific *Brassica* crops and their synergistic impact.

Biochar Materials	Microbes	Species	*Brassica* Crops	Synergistic Impact	Sources
Bamboo (*Bambusa* sp.)	Fungi	AMF	*Brassica oleracea* var. *alboglabra*	- Influenced plant growth, chlorophyll content, and photosynthetic pigment levels.	[20]
	Fungi	AMF	*B. napus*	- Promoted increases in plant height, biomass, leaf area, chlorophyll SPAD, antioxidant enzyme activity, leaf-soluble sugar content of *B. napus*; - Decreased cadmium (cd) and malondialdehyde (MDA)/hydrogen peroxide (H_2_O_2_) content.	[125]
Corncob (*Z. mays*)	Bacteria	*Ochrobactrum* sp. *Bacillus mucilaginosus*	*B. rapa*	- Promoted plant growth by increasing soil fertility; - Increased P and K contents in soil; - Increased P content in cabbage; - Enhanced crop growth and performance.	[122]
*Amaranthus*	Bacteria	*Serratia sp.* *Pseudomonas* sp.	*B. napus*	- Improved seed germination, plant biometrical (shoot and root biomass, length of organs) and physiological (photosynthetic pigments, proline, malondialdehyde, and relative water content) parameters under drought (exerted up to 50% of field capacity) and in Cd-spiked soil.	[19]
	Bacteria	*Enterobacter asburiae, Enterobacter tabaci,* and *Klebsiella variicola* in a 1:3:3 proportion	*B. chinensis*	- Enhanced soil enzyme activities (urease, catalase and phosphatase); - Increased activity involving heavy metals (HMs) (oxidizable, residual, and reducible); - Decreased acetic acid extraction; - Improved root and shoot length and root and shoot biomass; - Decreased content of Cd and Pb in the shoots of plants.	[126]
Rice (*O. sativa*) straw Rice (*O. sativa*) husks Soybean (*G. max*) straw Peanut (*A. hypogaea*) shells Corn (*Z. mays*) cobs Wood	Bacteria	*Arthrobacter defluvii* *Burkholderia cepacia* *Bacillus megaterium Pseudomonas frederiksbergensis Rhodanobacter* sp. *Streptomyces prasinopilosus* *Variovorax paradoxus*	*B. napus*	- Improved *B. napus* growth and P uptake.	[127]
Sugarcane bagasse (*S. officinarum*)	Bacteria	*Enterococcus gallinarum*	*B. napus*	- Enhanced physiological parameters in contrast to control P; - Enhanced growth and yield parameters (plant height, shoot fresh weight, shoot dry weight stem diameter, root length, root dry weight, and seed yield per pot).	[74]
Spent mushroom substrate	Bacteria	*Bacillus subtilis* *Pseudomonas fluorescence*	*B. oleracea* var. *botrytis*	- Improved the growth, yield, and biochemical parameters of plants (fresh plant biomass, dry plant biomass, plant height, root length, plant spread, and the number of leaves); - Best values for biochemical parameters and enzyme activities (total chlorophyll, superoxide dismutase, catalase, peroxidase, total phenolics, ascorbic acid, and total carotenoids);	[128]
Maize straw (*Z. mays*)	Bacteria	*Bacillus megaterium*	*B. chinensis*	- Increased *B. chinensis* growth; - Reduced Cd accumulation in plants; - Promoted uptake of P and N; - Recruited more plant growth-promoting bacteria in near-rhizosphere soil.	[18]
Paper and pulp waste	Bacteria	*Enterobacter* sp.	*B. napus*	- Effective in the amelioration of Cd stress; - Reduced Cd in soil, thereby decreasing its uptake in roots and shoots; - Improved shoot and root length, fresh and dry shoot and root weight, photosynthesis and transpiration rates, stomatal and sub-stomatal conductance, chlorophyll content, relative water content; - Decrease in electrolyte leakage, proline, malondialdehyde, catalase, glutathione peroxidase, glutathione S transferase, and superoxide dismutase.	[129]
Waste wood of *Morus alba*	Bacteria	*Pseudomonas* sp.	*B. napus*	- Supported plants to modify the efficiency of their water use, ultimately promoting growth; - Improved the canola plant defense system in terms of different antioxidant enzymes; - Improved plants’ drought tolerance.	[130]
Rice (*O. sativa*) husks	Bacteria	*Bacillus megaterium* *Serratia liquefaciens*	*B. campertris* ssp. *Chinensis* var. *Four-season*	- Decreased edible tissue metal uptake of vegetables by increasing pH, urease activity, amorphous iron (Fe) oxides, and *Leptothrix* species abundance in polluted soil.	[131]
Peach (*Prunus persica*) residues	Bacteria	*Pseudomonas putida* *Azotobacter chroococcum*	*B. napus*	- Improved root and shoot growth, total chlorophyll content, seed production, and oil yield of plants via alleviation of ionic and oxidative stresses by decreasing Na uptake, O_2_•‒, H_2_O_2_, and MDA content, increasing root and leaf nutrients, and stimulating oxidative defense by non-enzymatic antioxidants; - Enhanced root elongation and lateral root distribution; - Improved crop productivity under salinity via mitigating ionic and oxidative stresses and enhancing nutrients, antioxidant capacity, root and shoot growth, and chlorophyll content.	[132]
	Bacteria	*Azospirillum brasilense*	*B. chinensis*	- Decreased concentrations of Cd, nickel (Ni), lead (Pb), and zinc (Zn) in plants; - Decrease in the bioconcentration factor; - Increased biomass of the edible parts of plant; - Enhanced SPAD values; - Decreased MDA concentrations in stressed plants; - Increased soluble protein and sugar concentrations; - Improved flavonoids, total phenols, ascorbic acid, and DPPH levels.	[133]

## Data Availability

Data are contained within the article.
